# Are there reliable primary indicators of cardiorespiratory fitness in physically active students?

**DOI:** 10.1016/j.ijcrp.2026.200589

**Published:** 2026-02-04

**Authors:** Klaus Christian Haggenmüller, Sebastian Freilinger, Nils Olson, Jochen Weil, Thorsten Schulz, Renate Maria Oberhoffer, Barbara Reiner

**Affiliations:** a Chair of Preventive Pediatrics, Department of Health and Sport Science, School of Medicine and Health, Technical University of Munich, Am Olympiacampus 11, 80809, Munich, Germany; b German Heart Centre Munich, Hospital of the Technical University of Munich, Lazarettstraße 36, 80636, Munich, Germany

**Keywords:** Handgrip-strength, International physical activity questionnaire, Medical history interview, Cardio pulmonary exercise testing, Cardiovascular fitness

## Abstract

**Background:**

Cardiorespiratory fitness is a central component of preventive sports medical assessments and plays a crucial role in early identification of health risk factors in asymptomatic populations as well as evaluating physical performance. Preventive examinations in sports medicine frequently include the assessment of Handgrip-Strength (HGS), International Physical Activity Questionnaire (IPAQ), or Medical History Interviews (MHI). The aim of this study is to investigate whether one of these can serve as a valid surrogate marker for cardiorespiratory fitness.

**Methods:**

A total of 552 university students (mean age: 21.0 ± 3.79 years (37.5% male) underwent a standardized sports medical and motor performance assessment including Cardiopulmonary Exercise Testing (CPET) on a bicycle ergometer, IPAQ, HGS testing, and a structured MHI. Associations between VO_2_peak and the proposed surrogate markers were analysed, controlling for sex and body weight.

**Results:**

The parameters with the highest explanatory values were HGS (R = 0.439, p < .001) and MHI (R = 0.323, p < .001). The combination of these two parameters (HGS and MHI) had an explanatory value of R^2^ = 0.49 (F(3, 549) = 76.620, p < .001, n = 552).

**Conclusions:**

Maximal HGS and MHI showed potential as first-level indicators of cardiovascular fitness and in combination they may support screening in settings with limited resources and can be used for risk group identification. However, for medical applications the Cardiopulmonary Exercise Testing remains indispensable for an accurate and comprehensive assessment of individual cardiovascular fitness.

## 1 Introduction

Cardiorespiratory fitness is a central component of preventive sports medical assessments and plays a crucial role in early identification of health [1,2] risk factors in asymptomatic populations as well as evaluating physical performance. Within the context of preventive medical check-ups, the gold standard Cardiopulmonary Exercise Testing (CPET) is commonly used [3]. The primary outcome and predictor for cardiorespiratory fitness is the maximum peak of oxygen uptake VO_2_peak [4]. CPET requires significant time, financial resources, experienced professionals and specialized equipment [1].

Consequently, there is a growing interest in identifying alternative, easily applicable surrogate markers which are linked to VO_2_peak, especially in resource-limited settings. Numerous studies have investigated the associations between various testing methods for cardiopulmonary fitness in specific clinical populations [5,6]. Furthermore, a large study by Jackson et al. investigated a broad non-clinical population sample [7]. It remains unclear, if surrogate markers yield valid outcomes for a healthy sports related population.

Preventive examinations in sports medicine frequently include the assessment of one or more of the following tests, which could be linked to VO_2_peak: Handgrip-Strength Tests (HGS), the self-report International Physical Activity Questionnaire (IPAQ), and the responses from the medical history interview (MHI) regarding sports activity.

The aim of this study is to determine which of the three measurements HGS, the IPAQ, or MHI serves best as a valid surrogate marker for VO_2_peak in healthy, physically active university students.

## 2 Methods

This study is based on data from a preventive sports medical check-up program for first- and second-semester students enrolled in the bachelor's programs in sport and health sciences, which is part of TUM4Health, a university-based health promotion initiative. From May 2017 until July 2024, 552 students participated in this study (37.5% male). The students mean age was 21.0 ± 3.79 years. The male participants had an average height of 180.7 ± 7.55 cm and an average weight of 76.4 ± 12.86 kg. The female participants had an average height of 167.5 ± 6.18 cm and an average weight of 60.5 ± 8.27 kg (Table 1).

**Table 1:** Characteristics (HGS – Handgrip-Strength, MHI – Medical Health Interview, MET – Metabolic Equivalent of Task, vMet – vigorous Metabolic Equivalent of Task).

Characteristics	Total cohort	Male participants	Female participants	P value
	n = 552	n = 207	n = 345	
Age, years	21.0 ± 3.79	21.2 ± 4.27	21.0 ± 3.47	<0.001
Height, cm	172.4 ± 9.26	180.7 ± 7.55	167.5 ± 6.18	<0.001
Weight, kg	66.5 ± 12.83	76.4 ± 12.86	60.5 ± 8.27	<0.001
VO_2_peak, ml/min/kg	44.3 ± 7.82	49.8 ± 7.12	41.0 ± 6.25	<0.001
VO_2_peak, z score	1.4 ± 1.00	2.1 ± 0.91	1.0 ± 0.80	<0.001
HGS, kg/kg weight	0.6 ± 0.11	0.7 ± 0.10	0.5 ± 0.08	<0.001
HGS, z score	0.8 ± 1.00	1.0 ± 1.06	0.6 ± 0.87	<0.001
MET, h/week	72.1 ± 49.09	84.6 ± 49.49	64.8 ± 47.62	<0.001
MET, z score	1.3 ± 1.00	1.5 ± 1.00	1.1 ± 0.97	<0.001
vMET, h/week	39.6 ± 28.89	49.3 ± 34.33	33.6 ± 23.07	<0.001
MHI, h/week	6.6 ± 3.70	7.9 ± 3.79	5.8 ± 3.41	<0.001
MHI, z score	1.5 ± 1.00	1.8 ± 1.02	1.2 ± 0.93	<0.001

All voluntary participants were over 18 years old and gave their written informed consent. Ethical approval for the study was obtained from the Ethics Committee of the Technical University of Munich (Ref. 379/19 S-SR).

As bodyweight and sex are expected to have an influence on the results, relative values were used for statistical analysis. Therefore, the relative values per kg bodyweight were used (HGSmax/kg and VO_2_peak/ml/min/kg) and separated in male and female values where appropriate.

Other potential tests, such as maximal performance measures or submaximal prediction methods (e.g. step tests or cycle ergometer tests), were excluded due to their comparable time requirements to CPET and substantially higher costs compared to the selected methods.

Measurements were performed under standardized conditions to ensure reliability and consistency. The measurements were conducted by experienced personnel following a previously published protocol [8]. Examiners were supervised and retrained at regular intervals to ensure consistent application of the measurement procedure and to minimize inter- and intra-rater variability. The self-reporting questionnaires were filled out in presence of the examiners. Nevertheless, recall errors or social desirability could occur.

### 2.1 VO_2_peak

For the measurement of the VO_2_peak, a CPET was performed on a bicycle ergometer, using the ergometer Excalibur (Lode B.V., Netherlands). The ergometer was connected to the measuring device Metalyzer 3b (Cortex Biophysik GmbH, Germany) with an RS232 interface. While performing the CPET the participants were monitored by an ECG (12-channel-ECG Custo cardio 300). The blood pressure was measured with the automatic blood pressure measurement Suntech® Tango M2 (Cortex Biophysik GmbH, Germany) every 2 min. All devices were physically connected to the Metalyzer 3b, which used the „MetaSoft Studio“ software. This software was the control device for the examination process and configured individually to our workflow (Cortex Biophysik GmbH, Germany).

The protocol comprised 3 min at rest, a 3-min warm-up at 50 W, and a ramp phase starting at 50 W with increments of 20 W/min. Participants maintained a cadence of 60–80 rpm until exhaustion, followed by 3 min of recovery at 50 W with reduced cadence and 3 min at rest. Termination criteria were dyspnoea, dizziness/presyncope, angina pectoris, high stress extrasystoles, abnormal systolic blood pressure rise (>250 mmHg), or a significant systolic blood pressure drop during exercise.

The test results of the breath-by-breath-analysis were analysed using the “Wassermann 9 Panel Plot” [9]. The main outcome of this test is VO2peak. This is defined as the highest oxygen uptake achieved during the exercise test ([9], S. 158).

### 2.2 Handgrip strength

The HGS was measured with the MLT004/ST Grip Force Transducer (ADInstruments, New Zealand). The device was pre-calibrated and measured in the range of 0 to 800N with an accuracy of ± 5% [10]. The isometric dynamometer was connected with the PowerLab port from ADInstruments and a Lenovo PC with the software LabChart for Windows v.7.1. The HGS was measured according to a standardized protocol. The participants were instructed to stand in an upright position and flex the elbow joint of the test arm at 90°. The instructors were encouraging the participants. The participants performed two tries for each side, starting with the dominant hand [11]. The higher value of each side was noted. For further analysis the higher value from both sides was used. The output of the grip force device was recorded in Newton and was converted to kg (1 kg = 9.81 N).

### 2.3 International physical activity questionnaire

The participants were asked to fill out the IPAQ questionnaire online (in the short version). The questions referred to the amount of time spent per week on moderate- and high-intensity activities, as well as on walking. The analysis was following the guidelines of the IPAQ Research Committee, Version 2.April 0, 2004 [12]. In addition to the guidelines following analysis, the answers to vigorous activities were analysed (vigorous MET - vMET).

### 2.4 Medical history interview

As part of the medical history, the participants were asked how many hours of sports they participate during an average week. If there were any follow-up questions, it was clarified that the inquiry referred to all sporting activities, not regular daily activities. The weekly hours of sports were noted. The MHI is the value in hours of sports per week.

### 2.5 Data processing

Descriptive statistics and visualizations were generated using Microsoft Excel (Microsoft Corporation, Excel 2019, November 30, 2019). Subsequent statistical analyses were conducted with SPSS (IBM SPSS Statistics 29, Release November 13, 2022). No imputation procedures were applied, as only fully completed data were used for the analyses. A total of 27 datasets contained incomplete information (missing one or more examinations) and were therefore excluded from the evaluation. T-tests were used to compare group means. Linear correlations were examined using Pearson's and partial correlation coefficients [13]. Although some variables were not normally distributed, the large sample size (n > 339) allows robust parametric analyses based on the Central Limit Theorem [14]. With larger samples, parametric tests generally remain valid despite moderate deviations from normality [15]. Therefore, we considered the application of parametric methods appropriate for the present analysis. To analyse the relationship between a dependent and several independent variables, multiple regression models and regression equations [16] were used. Statistical significance was set at p < .05. Z-Scores were calculated to quantify the deviation of a measured value from an age- and sex-specific mean. Reference values for the CPET Z-Scores were taken from Rapp et al. [17]. Reference values for HGS Z-Scores, stratified by age and sex published by Steiber [18], were used. Data from the IPAQ were verified and processed according to the rules of the IPAQ Research Committee [12]. The WHO recommendation for physical activity in adults were used to evaluate the values of the study population and for Z-Scores [19].

## 3 Results

### 3.1 Test results

Participant characteristics are shown in Table 1. The average value for VO_2_peak was 44.3 ± 7.82 ml/min/kg, in the male subgroup 49.8 ± 7.12 ml/min/kg and 41.0 ± 6.25 ml/min/kg in the female. The age-related reference values for VO_2_peak in females was 33.5 ml/min/kg and for males VO_2_peak = 37.7 ml/min/kg [17]. The Z-Scores for the whole population were 1.4 ± 1.00, in the male subgroup 2.1 ± 0.91 and 1.0 ± 0.80 in the female. All values reached statistical significance.

The average value for HGS by kg bodyweight was 0.6 ± 0.11 kg. For the males 0.7 ± 0.10 kg, and for the females 0.5 ± 0.08 kg. The reference value for HGS for females was in average 0.49 kg (HGS average 32.5 kg, size 165 to 169 cm, weight 66.0 kg) and for males HGS = 0.59 kg (HGS average 51.2 kg, size 180 to 184 cm, weight 85.8 kg) [18]. The Z-Scores were 0.8 ± 1.00 and for the male subgroup 1.0 ± 1.06, respectively 0.6 ± 0.87 for the females. All values reached statistical significance.

The average value for MET was 72.1 ± 49.09 MET/h/week, for the males 84.6 ± 49.49 MET/h/week, and for the females 64.8 ± 47.62 MET/h/week. The WHO recommendation for physical activity in adults is 2.5 h low intense training or 1.25 h high intense training [20]. In MET this would be 10 MET (2.5 h ∗ 4 or 1.25 h ∗ 8). As shown in the Eurobarometer Study [21,22], which also used the IPAQ, 40.2% of the German population fulfilled this recommendation, with 91% in the cohort of this study, was clearly higher. Correspondingly the Z-Scores were 1.3 ± 1.00 for the total cohort, 1.5 ± 1.00 in the male subgroup and 1.1 ± 0.97 in the female. For the vigorous MET (vMET/h/week) the average was 39.6 ± 28.89, in the male subgroup 49.3 ± 34.33 and in the female 33.6 ± 23.07. All values reached statistical significance.

The average value for hours spent in sports per week (MHI) was 6.6 ± 3.70 h, in the male subgroup 7.9 ± 3.70 h, and for the females 5.8 ± 3.41 h. The WHO recommendation for high physical activity in adults is more than 1.25 h high intense training per week [19] In our total cohort 98% fulfilled the WHO recommendation. The Z-Scores were 1.5 ± 1.00 for the total cohort, 1.8 ± 1.02 in the male subgroup and 1.2 ± 0.93 in the female. All values reached statistical significance.

### 3.2 Bivariate correlations

**Fig. 1:**
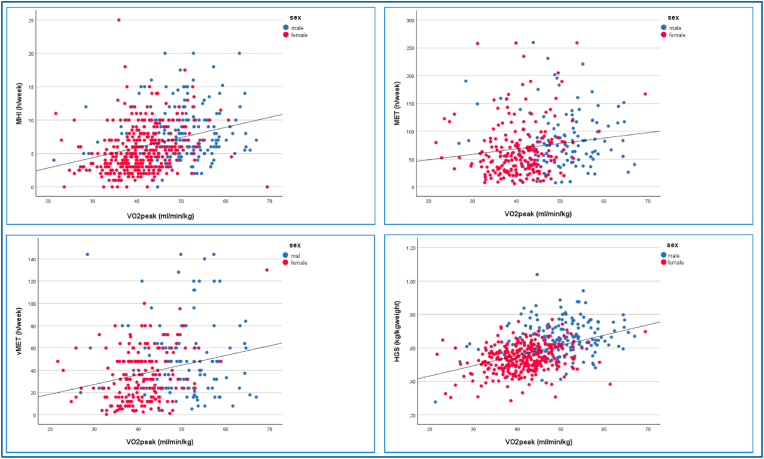
Visualization of correlations between Medical History Interview (MHI), Handgrip Strength (HGS), Metabolic Equivalent of Task (MET), vigorous Metabolic Equivalent of Task (vMET) and maximum peak of oxygen uptake (VO2 peak).

The Bivariate correlations values are shown in Table 2 and the distribution in Fig. 1. VO_2_peak was significant positively correlated with HGS R = 0.439. In the female subgroup the correlation was significant R = 0.278. The vigorous vMET values were significant with R = 0.250 for the total cohort and, R = 0.301 in the female subgroup. The significance of the MHI values were similar to vMET correlation of R = 0.323 for the total cohort, and R = 0.291 in the females.

**Table 2:** Univariable correlations.

Bivariate Correlations to VO2peak (ml/min/kg)	Total cohort		Male participants		Female participants	
	n = 552		n = 207		n = 345	
	R	p	R	p	R	p
HGSmax, kg/kg weight	0.439	<0.001	0.131	0.080	0.278	<0.001
MET, h/week	0.160	0.003	0.051	0.575	0.034	0.034
vMET, h/week	0.250	<0.001	0.141	0.676	0.301	<0.001
MHI, h/week	0.323	<0.001	0.123	0.081	0.291	<0.001

### 3.3 Multiple regression model

**Fig. 2:**
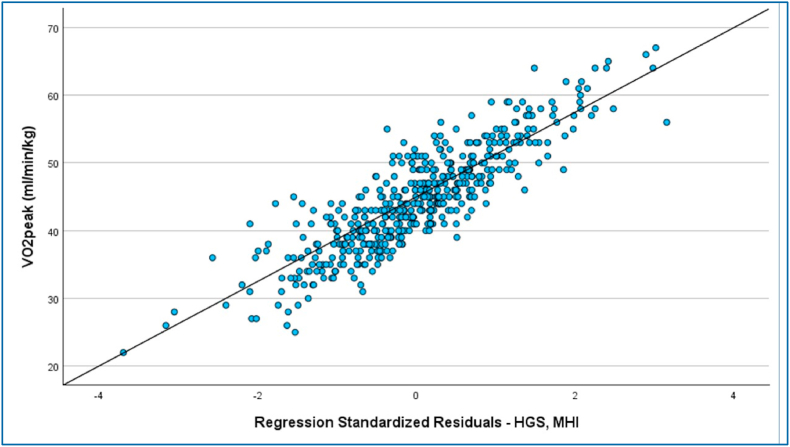
Combined regression model handgrip strength (HGS) and medical history interview (MHI).

The results from the multiple regression (covariates sex and weight) model are shown in Fig. 2. The figure illustrates the relationship between VO_2_peak and four normalized parameters - MET, vMET, HGS, and MHI - with all values adjusted for body weight. Individual data points are shown, with male participants represented in blue and female participants in red. A correlation line is included in each plot to indicate the strength and direction of the relationship between VO_2_peak and the respective parameter. This visualization allows for assessment of both the overall trend and the distribution of individual measurements across sexes. The corresponding regression equations are listed in the additional material (Table 3). For the HGS-model the explanatory value of HGS was R^2^ = 0.196, HGS (F(3, 549) = 71.724, p < .001, n = 552). The MET-model had an R^2^ = 0.026, MET (F(3, 549) = 52.234, p < .001, n = 552). The vMET-model had an R^2^ = 0.062, vMET (F(3, 549) = 45.103, p < .001, n = 552). In the MHI-model the values were R^2^ = 0.103, VO_2_peak = 1.988 + 0.894∗sex +0.19∗weight +0.035∗MHI.

**Table 3:** Multiple regression equations.

y	b_0_	b_1_x_1_	b_2_x_2_	b_3_x_3_
VO_2_peak	1.886	0.815 sex	0.017 wt	0.011 HGS
VO_2_peak	2.102	0.993 sex	0.210 wt	0.001 MET
VO_2_peak	2.286	0.995 sex	0.180 wt	0.030 vMET
VO_2_peak	1.988	0.894 sex	0.190 wt	0.035 MHI

The parameters with the highest explanatory values were HGS and MHI. The combined model is shown in Fig. 2. The figure illustrates the combined regression model of HGS and MHI. Each data point is represented as a circle, and the regression line (fitted regression curve) shows the relationship between the combined HGS–MHI score and VO_2_peak. The explanatory value was R^2^ = 0.49 (F(3, 549) = 76.620, p < .001, n = 552) with an equation of VO_2_peak = 25.752 + 0.375∗HGS+0.254∗MHI.

## 4 Discussion

The capability of the analysed markers in this cohort was different. Despite the association of VO_2_peak to all markers, the correlation and explanatory value of single markers was not high enough to serve as a surrogate in a sport-cohort. However, the combination of HGS and MHI worked best and has the potential to be used for first-level analysis of cardiorespiratory capability.

The aim of this study was to investigate whether easy-to-use and cost-effective primary indicators of cardiovascular fitness exist for a sports related population. The selected markers met the criteria of cost-efficiency, easy to use and regularly included in sports medical examinations. The cohort of this study was noticeably sports related. The Z-Scores of the sports marker were clearly above normal population values. Especially the endurance Scores for VO_2_peak and vMET were high above references. The resistance Z-Scores HGS and the marker for all daily movements MET were also elevated.

HGS had the strongest association with VO_2_peak among all markers, although the correlation was moderate. This is in line with literature, where HGS has been confirmed in numerous studies as a possible marker for CV fitness. However, the effect size and strength of the association was evaluated differently across studies [[23], [24], [25]]. Nevertheless, it might serve as a first level indicator especially in combination with others. The correlation of HGS varied between male and female subgroups, with HGS showing stronger associations within females. From a physiological perspective this could be related to the differences in muscle mass between the groups [26,27]. Moreover, behavioral factors like differences in trainings patterns could be responsible for the less significant correlation in men [28,29]. Especially males doing isolated strength training (e.g., weightlifting), which increases HGS without necessarily improving aerobic fitness [30].

In contrast to other questionnaires on physical activity, the MHI was notably straightforward, and the correlations were on second place. This approach provided a clear timeline for recalling physical activity in the long run. However, the correlation remained moderate. This result is in line with the study by Jackson et al. who concluded associations between cardiovascular fitness and reported physical activity [7].

MET was only low correlated with VO_2_peak, this is in line with previous publications. Van Poppel et al. published a systematic review and also found medium and low correlations between MET calculated from the IPAQ questionnaire and accelerometer data [31]. The MET combines low-cardio and high-cardio elements. Therefore the analysis was repeated based on the vigorous activity parameter. The correlations between vMET and VO_2_peak were higher than the MET values, but still on a low level. In summary, while HGS and MHI cannot replace CPET in clinical assessment, they provide practical first-level tools for large-scale screening and risk group identification, particularly in resource-limited settings. The IPAQ possibly is not sensitive enough to be used for athletes or highly active populations, due to few classifications of intense activities [32]. Objective measures (e.g., wearable activity trackers) might yield more reliable results.

The conclusions apply to healthy, physically active young adults; therefore, the findings cannot be generalized to the general population or to individuals with low fitness levels. A restriction of the homogeneous study sample is a reduced variability, limiting the external validity of the findings. Future studies could involve more heterogeneous and less sport-oriented populations, to prove the results for different populations.

The selected tests were chosen due to their cost-effectiveness and use in sports medical settings. In future studies, the use of submaximal prediction methods, such as step tests or cycle ergometer protocols like the Modified Canadian Aerobic Fitness Test could serve as a potential compromise between simplicity and physiological relevance. Moreover, the cross-sectional approach provides valuable insights into relevant associations and serves as a foundation for future studies aimed at investigating causality and predictive validity longitudinally. Building on the present findings, subsequent studies may benefit from applying a longitudinal design to assess the stability of the identified indicators, their prognostic value, and their relationship with health outcomes. Furthermore, the inclusion of additional relevant variables beyond sex and body weight - such as smoking behaviour, dietary habits, sleep quality, medication use, and alcohol consumption - could enhance the explanatory power and comprehensiveness of future analyses.

## 5 Conclusion

Maximal HGS and MHI showed potential as first-level indicators of cardiovascular fitness for young and active young adults and in combination they may support screening in settings with limited resources and can be used for risk group identification. However, for medical applications the Cardiopulmonary Exercise Testing, remains indispensable for an accurate and comprehensive assessment of individual cardiovascular fitness. The practical utility of the combined HGS and MHI is restricted to use in preliminary screening procedures and does not extend to diagnostic assessments. Future research in more diverse populations is needed to establish robust, clinically meaningful cut-offs. Moreover, sex-specific screening strategies should be further evaluated.

## CRediT authorship contribution statement

**Klaus Christian Haggenmüller:** Writing – original draft, Visualization, Conceptualization. **Sebastian Freilinger:** Writing – review & editing, Formal analysis. **Nils Olson:** Writing – review & editing. **Jochen Weil:** Writing – review & editing, Validation, Conceptualization. **Thorsten Schulz:** Writing – review & editing, Supervision, Project administration. **Renate Maria Oberhoffer:** Writing – review & editing, Project administration, Conceptualization. **Barbara Reiner:** Writing – review & editing, Supervision, Project administration, Conceptualization.
